# Intrafractional stability of MR-guided online adaptive SBRT for prostate cancer

**DOI:** 10.1186/s13014-021-01916-0

**Published:** 2021-09-26

**Authors:** J. Schaule, M. Chamberlain, L. Wilke, M. Baumgartl, J. Krayenbühl, M. Zamburlini, M. Mayinger, N. Andratschke, S. Tanadini-Lang, M. Guckenberger

**Affiliations:** Department of Radiation Oncology, University Hospital Zurich, Rämistrasse 100, 8091 Zurich, Switzerland

**Keywords:** MR-guided radiotherapy, Online adaptation, Prostate SBRT, Plan-of-the-day

## Abstract

**Background:**

MR-guided online adaptive stereotactic body radiation therapy (SBRT) for prostate cancer aims to reduce toxicity by full compensation of interfractional uncertainties. However, the process of online adaptation currently takes approximately 45 min during which intrafractional movements remain unaccounted for. This study aims to analyze the dosimetric benefit of online adaptation and to evaluate its robustness over the duration of one treatment fraction.

**Methods:**

Baseline MR-scans at a MR-linear accelerator were acquired for ten healthy male volunteers for generation of mock-prostate SBRT plans with a dose prescription of 5 × 7.25 Gy. On a separate day, online MR-guided adaptation (ViewRay^®^ MRIdian) was performed, and thereafter MR images were acquired every 15 min for 1 h to assess the stability of the adapted plan.

**Results:**

A dosimetric benefit of online MR-guided adaptive re-planning was observed in 90% of volunteers. The median D_95_CTV- and D_95_PTV-coverage was improved from 34.8 to 35.5 Gy and from 30.7 to 34.6 Gy, respectively. Improved target coverage was not associated with higher dose to the organs at risk, most importantly the rectum (median D_1cc_rectum baseline plan vs. adapted plan 33.3 Gy vs. 32.3 Gy). The benefit of online adaptation remained stable over 45 min for all volunteers. However, at 60 min, CTV-coverage was below a threshold of 32.5 Gy in 30% of volunteers (30.6 Gy, 32.0 Gy, 32.3 Gy).

**Conclusion:**

The dosimetric benefit of MR-guided online adaptation for prostate SBRT was robust over 45 min in all volunteers. However, intrafractional uncertainties became dosimetrically relevant at 60 min and we therefore recommend verification imaging before delivery of MR-guided online adapted SBRT.

**Supplementary Information:**

The online version contains supplementary material available at 10.1186/s13014-021-01916-0.

## Introduction

Stereotactic body radiotherapy (SBRT) has been implemented in the treatment of localized prostate cancer and evaluated in multiple prospective phase II trials, which have shown comparable outcomes to other treatment modalities both, with regard to toxicities, and to long-term recurrence-free survival [1–4]. Consequently, SBRT today represents an alternative to conventionally fractionated radiotherapy for low to intermediate risk prostate cancer at clinics with appropriate technology, physics, and clinical expertise according to the NCCN guidelines v2.2020 [5–7].

With higher doses per fraction comes the necessity of accurate dose-delivery, conformal to the target and adjusted to the changing anatomy of the small pelvis. Magnetic resonance (MR)-guided radiotherapy with daily adaptation has the potential to improve the precision and accuracy of radiotherapy through continuous tracking of the target volume and image-guidance with improved soft-tissue contrast and anatomic visualization compared to cone-beam computer tomography (CBCT) scans [8]. Daily online plan-adaptation can yield reduced planning target volume (PTV) margins by accounting for movements in both, the clinical target volume (CTV) and organs at risk (OAR) in close proximity. It promises to compensate for both, systematic anatomic changes, like prostate swelling, as well as random changes, such as inter- and intrafractional rectal and bladder fillings.

Prior to the availability of MR-guided online adaptation, stereotactic treatment plans for prostate cancer were not routinely adapted online, mainly due to the length of the planning process and the insufficient soft tissue contrast of CBCT. MR-guided linear accelerators are now commercially available offering continuous visualization and online adaptation. However, the workflow to perform this adaptation has been reported to be in the range of 50 min, which is in agreement with our own experiences [9–12].

During this time, bladder filling, peristalsis and air passing through the rectum may be sources of significant changes in the immediate anatomy of the prostate. The aim of this study was therefore to analyze the dosimetric benefit of performing an MR-guided online adaptation in SBRT for prostate cancer and to elucidate its stability.

## Methods

Ten healthy male volunteers (27–51 years of age, median 35) were recruited and scanned in the ViewRay^®^ MR-linear accelerator (ViewRay^®^ Inc., Mountain View, CA) at the University Hospital Zurich at two different timepoints. All volunteers had previously consented to participate in this study. Ethical review and approval was acquired from the cantonal ethics committee Zurich (2021-00158).

For immobilization, a headrest and a Knee-Fix were used. On day one, volunteers were positioned for a mock prostate SBRT treatment in the MR-Linac. Set-up images were acquired in low resolution, and the couch was shifted to the expected isocenter located within the prostate. A 3D-simulation high-resolution MR-scan (with 0.15 cm resolution during a 128–173-s image acquisition with a field of view 40 × 43 × 40) was acquired in free breathing. True FISP (fast imaging with steady state procession) sequences were acquired using balanced gradients. A baseline prostate SBRT plan was created according to the workflow described below.

On a different day, the volunteers were positioned identically in the MR-Linac and another low-resolution MR-scan was acquired for set-up. The prostate position was registered in AP, LR and SI direction between the baseline scan and the verification scan to compensate for interfractional translational shifts of the prostate position, and the couch was shifted accordingly. By correcting these translation shifts, we were able to independently analyze the effect of rotational errors as well as deformations of the prostate. The baseline SBRT plan was copied onto the first MR of the day and was subsequently optimized to create the plan-of-the-day using identical IMRT planning objectives as at baseline. Over 1 h, we acquired additional high-resolution MR scans in 15 min intervals in treatment position to analyze the stability of the adapted plan over these 60 min. For every consecutive MR-scan, a registration of the prostate in translational AP, LR and SI directions was performed, and the couch was shifted accordingly. The shifts are shown in Additional file 1: Table S1. The adapted plan was then copied onto the consecutive MR-scans and was recalculated on the anatomy from the consecutive MR-scans obtained after 15, 30, 45, and 60 min (MR2-5).

**Fig. 1:**
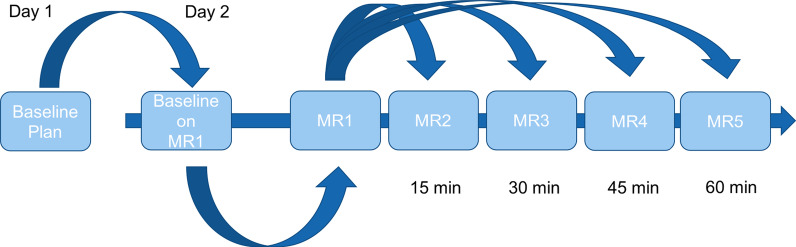
The workflow

The target volume included the healthy prostate and the lower third of the seminal vesicles, which were contoured by one physician (JS) using MIMvista software as the clinical target volume (CTV), and a margin of 5 mm (3 mm posteriorly towards the rectum) was added to form the planning target volume (PTV). OAR including the entire rectum (not rectum wall), entire bladder, penile bulb, and femur heads were contoured for all volunteers at all timepoints. Contours were propagated and adjusted to changes of volume and shape over time. The workflow is outlined in Fig. 1. Of note, we abstained from bladder or rectum filling regimens, and folly catheters for the healthy volunteers.

### Fractionation, plan evaluation and analysis

A dose of 36.25 Gy in 5 fractions (equal to an estimated 2 Gy-equivalent dose of 90.5 Gy for an alpha/beta value of 1.5) was prescribed to the planning target volume to cover 95% of the PTV with 95% of the prescribed dose. PTV and OAR constraints are shown in Additional file 2: Table S2. We deemed a threshold of 32.5 Gy to be the minimally acceptable D_95_CTV according to Kishan et al. as their study represented a similar dosing regimen to this study [1]. 32.5 Gy in 5 fractions is equivalent to an EQD2 dose of 74 Gy, assuming an alpha/beta value of 1.5. This dose is considered as the minimum dose in a curative setting in conventional fractionation.

A 9-field step-and-shoot IMRT plan was calculated with the ViewRay^®^ Planning System using 6 MV flattening filter free photons with a maximum dose rate of 600 MU/min; dose calculation used Monte Carlo with a statistical uncertainty of 1% and grid spacing of 3 mm. All plans were verified with the MR-compatible Delta 4 Phantom (ScandiDos, Uppsala, Sweden) and passed with a gamma criterion of 3% 3 mm with a passing rate above 95%.

In order to compare the different plans, all treatment plans were transferred into MIM-software^®^ (MIM-software^®^ Inc., Cleveland, OH) and dose volume histograms were exported for evaluation.

Statistical analyses were performed with GraphPad Prism software version 8.2.0. Distribution of data was assumed to be non-Gaussian and therefore only non-parametric tests were applied. For comparing the difference in absolute dose to different targets, Wilcoxon matched pairs signed rank test was used. A *p* value of 0.05 was deemed to be statistically significant.

## Results

### Online adaptive re-planning

**Fig. 2:**
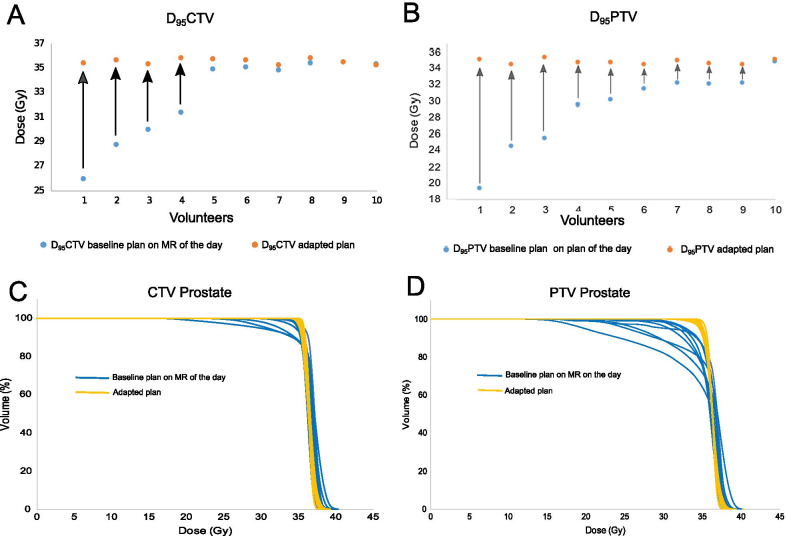
Dosimetric benefit of adaptation compared to original plan **A**, **B** median D_95_CTV and D_95_PTV comparison of absolute doses with blue = baseline plan and orange = adapted plan **C**, **D** dose volume histograms for D_95_CTV and D_95_PTV for blue = baseline plan and orange = adapted plan

Image quality of the MR-scans was deemed sufficient for baseline planning and adaptive re-planning in all volunteers and at all timepoints by a highly experienced medical physicist and a radiation oncologist experienced in MR-based prostate delineation. Both, CTV- and PTV-coverage were improved by MR-guided online adaptation in 90% of volunteers (Fig. 2 baseline vs. adapted plan D_95_CTV, Table 1).

**Table 1:** Comparison of absolute dose (Gy) for organs at risk and coverage for the baseline plan on the MR of the day versus the adapted plan for ten volunteers

	Baseline plan on MR of the day				Adapted plan			
	Median	Mean	Max	Min	Median	Mean	Max	Min
D_1cc_rectum	33.3	31.6	37.0	21.3	32.3	33.0	35.5	30.7
D_mean_bladder	9.8	12.0	19.8	3.3	10.1	11.9	20.1	3.4
D_95_PTV	30.7	29.1	34.7	19.3	34.6	34.7	35.2	34.4
D_95_CTV	34.8	32.6	35.4	25.9	35.5	35.6	35.3	35.1

Ten baseline plans of the day were compared with ten adapted plans, respectively

The median D_95_CTV- and D_95_PTV-coverage was improved from 34.8 to 35.5 Gy (*p* = 0.006) and 31.4 to 34.6 Gy (*p* = 0.005), respectively. Online adaptive re-planning with recovery of target coverage was not associated with higher doses to the OAR, most importantly the rectum (Additional file 3: Fig. S1). The median D_1cc_rectum was lower in the adapted plans compared to the baseline plan copied onto the MR of the day with 33.33 Gy versus 32.3 Gy. Similar results were observed for the D_mean_bladder (9.6 Gy vs. 8.8 Gy, Table 1).

### Stability of online adapted SBRT plans

Repetitive MR-scans were acquired for ten volunteers in 15 min intervals for a total duration of 60 min. There was no systematic shift in any direction and at any timepoint larger than 2 mm. The 3D vectors of intrafractional prostate drifts at timepoints 15, 30, 45 and 60 min were 1.2 mm, 1.6 mm, 1.0 mm and 0.4 mm, respectively. Full shifting data is displayed in Additional file 1: Table S1.

**Fig. 3:**
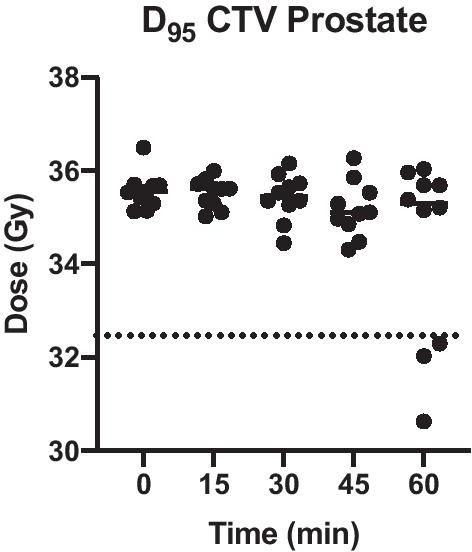
Absolute D_95_CTV of adapted plan over 1 h in 15 min intervals for ten volunteers. The bars depict the median dose. The dotted line represents the minimally acceptable D_95_CTV threshold dose

**Fig. 4:**
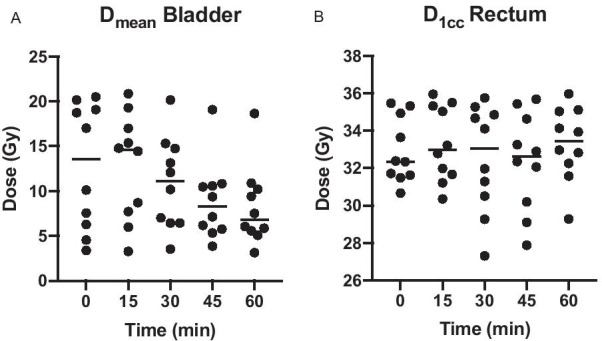
**A** D_mean_bladder and **B** D_1cc_rectum of ten volunteers over 1 h in 15 min intervals. The bars depict the median dose

For assessment of recalculated plans at 15, 30, 45 and 60 min, we defined a threshold of 32.5 Gy as the minimally acceptable D_95_CTV, which represents 74 Gy EQD_2Gy_ assuming an alpha/beta value of 1.5 Gy [13]. Consider that all translational drifts of the prostate have been corrected before dose re-calculation. The median D_95_CTV remained stable (0 min: 35.5 Gy, 60 min: 35.3 Gy) and the difference compared to baseline remained statistically non-significant for all timepoints (*p* = 0.26 for timepoint 0 vs. timepoint 60 min). Target coverage remained above this threshold for all ten volunteers at timepoints 15, 30 and 45 min (Fig. 3). At 60 min, three volunteers had an unacceptable D_95_CTV below the threshold of 32.5 Gy due to non-rigid anatomical deformations. With respect to doses to OAR, the D_mean_bladder decreased significantly over the one-hour time period due to increasing filling (Fig. 4A *p* = 0.01 timepoint 0 vs. timepoint 60 min). The D_1cc_rectum did not differ between timepoint 0 and 60 min (Fig. 4B *p* = 0.59).

## Discussion

This analysis shows that MR-guided online adaptation of SBRT for prostate cancer achieves a dosimetric benefit that is stable over the time currently required for this complex and multi-professional process and may yield clinical benefits. However, caution should be taken, because larger non-rigid intrafractional variations were observed in few volunteers, which resulted in decreasing target coverage at 60 min. We therefore recommend a verification MR-scan after the plan adaption process and before SBRT delivery to assess stability of the adapted treatment plan as a standard procedure.

We showed that a better target coverage was not associated with higher dose to the OAR, most importantly the rectum. Here, we would like to stress that we delineated the entire organ and not just the rectum and bladder wall as an OAR and therefore hotspots could have been located in the lumen not contributing to possible clinical toxicity. In future, delineation of rectum or bladder wall may be considered in MR-based planning.

To date, the stability of MR-guided adaptation benefit for prostate SBRT over time has only been investigated in a small number of patients (n = 5) treated with 20 × 3.1 Gy by de Muinck Keizer et al. [14]. During the on-couch period of 45 min in accordance with our assumptions, the group showed that the variance of intrafraction motions grows over time but that margins do not need to be increased beyond 5 mm. The group had formerly investigated intrafractional motion during prostate MR-guided SBRT using four implanted gold fiducial markers [15]. Here, they reported movements of the markers at ten minutes of X: 0.0 ± 0.8 mm; Y: 1.0 ± 1.9 mm and Z: 0.9 ± 2 mm, as well as mean rotation of X: 0.1 ± 3.0°, Y: 0.0 ± 1.3° and Z: 0.1 ± 1.2°. However, the dosimetric consequences were not analyzed and the time frame was rather short with ten minutes. Before MR-guided radiotherapy was available, inter- and intrafractional motion has been described using electromagnetic transponders (Calypso^®^ system) and gold markers [16–23]. In these studies, maximal mean interfractional motion of the prostate varied between 3.1 and 5 mm. For intrafractional motion, studies showed both, persistent drifts and rapid movements with varying reports of > 5 mm deviations in 15% of cases to intrafractional changes of < 1 mm in all cases.

Real-time tracking and adaptation has recently become semi-automated as an algorithm proposed by Olsen et al. shows [24]. The authors have described a method that reduced the workload of adaptive planning and included compensation for both, translational and rotational movements—however the authors point out that fiducial geometry and axis of rotation has large impact on interpreting tracking data. While this algorithm is able to facilitate tracking, implanting the required markers remains an invasive method, which poses interventional risks such as bleeding and infection, possibly superfluous with this advanced image guidance.

A recent phase III study comparing normofractionated, hypofractionated and stereotactic radiotherapy for low to intermediate risk prostate cancer showed no increase in toxicity for SBRT compared to hypofractionation, although long-term data is still lacking [25]. However, image-guidance and adaptive planning may yield clinical relevance through reducing margins in light of the development towards even more extreme hypofractionation which are associated with higher risk for toxicities. A recent phase I dose escalation trial concluded that 36 Gy in only 4 fractions (with a 2-day break between fractions) prescribed at the 95% isodose level is recommendable with an acceptable toxicity profile [26]. Of note, the authors did not report the image-guidance that was available to them for delivery of single doses of 8–9 Gy. Potter and colleagues investigated delivering up to 50 Gy in 5 fractions using a Calypso-based system and kV and CBCT-image guidance in a phase I trial with an improved PSA-nadir for 45–50 Gy total dose without higher incidence of grade 3 toxicity [27]. Studies evaluating even single-fraction SBRT are currently enrolling patients [28].

Multiple treatment regimens for all stages of prostate cancer are currently vigorously examined by a multitude of phase I–III trials (One-Shot NCT03294889, PRIME NCT03561961, PATRIOT [29] and others). First clinical experience shows favorable quality of life for patients treated with MR-guided SBRT for prostate cancer [12].

All new hypofractionated treatment regimens have in common the need for daily imaging to minimize toxicity and avoid underdosing of target volumes highlighting the future utility of MR-guided online adaptation systems.

The limitations of this study include its single center character, the relatively small sample size, the omission of a bladder or rectum protocol, as well as Foley catheters in this set of volunteers that was younger than the usual patient cohort.

A bladder and rectum protocol, as well as Foley catheters were omitted, because albeit side effects of these interventions are rare, we did not wish to expose the healthy volunteers to any risks. We acknowledge, that this might present a deviation from clinical practice; however, the clinical benefit of a bladder and rectum preparation protocol remains uncertain and has not been validated in randomized trials [30, 31]. From an anatomical and dosimetric perspective, rectum filling and bladder filling do influence the doses delivered to these organs at risk. An empty rectum and a full bladder would be optimal for OAR sparing. However, the reproducibility especially of a filled bladder during a course of fractionated radiotherapy is an issue of concern.

Importantly, the volunteers were younger (median age 35 years) than the average prostate cancer patient, which may result in higher compliance and fewer internal anatomical shifts. While constipation or flatulence might be more common in an older age group (although data supporting this are missing), in clinical practice, the bladder or rectum protocol would be in place to at least alleviate the effects. Due to the possibility of target and OAR shifts over time, it is of utmost importance to perform imaging and treatment as promptly as is safely possible.

This study does not unequivocally imply a clinical benefit for patients. The small sham trial was not designed to prove such clinical benefit. The study does, however, show dosimetric benefits that may translate to clinical benefits. Larger studies designed to show a clinical benefit will need to examine whether that hypothesis holds true.

In conclusion, when performing prostate SBRT, daily imaging is of utmost importance due to interfractional anatomic variability. Daily plan-adaptation yields significant dosimetric and potentially clinically relevant benefits, that are stable over at least 45 min. Crucially, outliers exist with large intrafractional deformations and given the lack of markers to identify these movers prospectively, a MR verification scan before dose-delivery is highly recommended to ensure exact treatment. In the future, faster and possibly (semi-) automated adaptation workflows, or continuous imaging could speed up the adaptation process, reducing the relevance of intrafractional motion during adaptation.

## Supplementary Information

**Additional file 1: Table S1.** Couch shifts; A lateral, B vertical, C axial shifts in cm. Each line represents one volunteer. Bold = median.

**Additional file 2: Table S2.** Dose Constraints.

**Additional file 3: Fig. S1.** D_1cc_rectum for the baseline plan on the MR of the day compared to the adapted plan. Bar = median.

## Data Availability

The dataset(s) supporting the conclusions of this article is(are) included within the article (and its additional file(s)).
