# Supplementary material for: Intrafractional stability of MR-guided online adaptive SBRT for prostate cancer
Source: Radiat Oncol. 2021 Sep 26;16:189. doi: 10.1186/s13014-021-01916-0 (PMC8474766; doi:10.1186/s13014-021-01916-0)
Supplement: Supplementary file 1 — Additional file 1: Table S1. Couch shifts; A lateral, B vertical, C axial shifts in cm. Each line represents one volunteer. Bold = median. [file 13014_2021_1916_MOESM1_ESM.docx]

Additional Table 1: Couch shifts; A lateral, B vertical, C axial shifts, D 3D-vector shifts in cm. Each line represents one volunteer. Bold = median.

**A** lateral:

| MR2 | MR3 | MR4 | MR5 |
| --- | --- | --- | --- |
| -0.1 | 0.15 | -0.05 | 0 |
| 0.13 | -0.01 | 0.02 | -0.05 |
| 0 | 0.1 | 0 | 0 |
| 0 | 0 | 0 | 0 |
| -0.7 | 0 | 0 | 0 |
| 0 | 0 | 0.01 | 0 |
| -0.1 | 0.1 | 0.05 | 0 |
| 0.1 | 0 | 0 | 0 |
| -0.3 | -0.15 | 0 | 0 |
| 0 | 0.05 | -0.1 | 0 |
| **0** | **0** | **0** | **0** |

**B** vertical:

| \| MR2 \| MR3 \| MR4 \| MR5 \| \| --- \| --- \| --- \| --- \| \| 0.1 \| 0.25 \| 0.1 \| 0.15 \| \| -0.27 \| 0.09 \| -0.02 \| 0.1 \| \| 0.1 \| 0.05 \| -0.85 \| 0 \| \| -0.2 \| 0 \| 1.85 \| 0 \| \| 0.05 \| 0.14 \| 0 \| 0 \| \| 0.25 \| -0.3 \| 0.25 \| 0 \| \| 0.1 \| 0.55 \| -0.4 \| 0 \| \| -0.6 \| 0.95 \| 0 \| 0 \| \| -0.2 \| -0.1 \| -0.15 \| 0 \| \| 0 \| -0.1 \| 0.1 \| 0 \| \| **0.025** \| **0.07** \| **0** \| **0** \| |  |  |  |  |
| --- | --- | --- | --- | --- | --- | --- | --- | --- | --- | --- | --- | --- | --- | --- | --- | --- | --- | --- | --- | --- | --- | --- | --- | --- | --- | --- | --- | --- | --- | --- | --- | --- | --- | --- | --- | --- | --- | --- | --- | --- | --- | --- | --- | --- | --- | --- | --- | --- | --- | --- | --- | --- |

**C** axial:

| MR2 | MR3 | MR4 | MR5 |
| --- | --- | --- | --- |
| -0.33 | 0.05 | 0 | 0 |
| 0.07 | 0.08 | -0.02 | 0.06 |
| 0 | 0.15 | -0.7 | 0 |
| -0.4 | 0 | 0.11 | 0 |
| 0.42 | 0.08 | 0 | 0 |
| 0.1 | -0.15 | 0.26 | 0 |
| 0.25 | 0.4 | 0 | 0.1 |
| -0.45 | 0 | 0 | 0 |
| 0.2 | 0 | 0.7 | 0 |
| 0.05 | -0.05 | 0.1 | 0 |
| **0.06** | **0.025** | **0** | **0** |

**D** 3D-vectors:

|  | 15 mins | 30mins | 45mins | 60mins |
| --- | --- | --- | --- | --- |
| Volunteer 1 | 0.36 | 0.30 | 0.11 | 0.15 |
| Volunteer 2 | 0.31 | 0.12 | 0.03 | 0.13 |
| Volunteer 3 | 0.10 | 0.19 | 1.10 | 0.00 |
| Volunteer 4 | 0.45 | 0.00 | 1.85 | 0.00 |
| Volunteer 5 | 0.82 | 0.16 | 0.00 | 0.00 |
| Volunteer 6 | 0.27 | 0.34 | 0.36 | 0.00 |
| Volunteer 7 | 0.29 | 0.69 | 0.40 | 0.10 |
| Volunteer 8 | 0.76 | 0.95 | 0.00 | 0.00 |
| Volunteer 9 | 0.41 | 0.18 | 0.72 | 0.00 |
| Volunteer 10 | 0.05 | 0.12 | 0.17 | 0.00 |
