# Supplementary material for: Intrafractional stability of MR-guided online adaptive SBRT for prostate cancer
Source: Radiat Oncol. 2021 Sep 26;16:189. doi: 10.1186/s13014-021-01916-0 (PMC8474766; doi:10.1186/s13014-021-01916-0)
Supplement: Supplementary file 2 — Additional file 2: Table S2. Dose Constraints. [file 13014_2021_1916_MOESM2_ESM.docx]

Additional Table 2: Dose Constraints

| **Targets / OAR** | **Prescription Constraint** |
| --- | --- |
| PTV | D_95_ ≥ 34.4Gy  D_0.1cc_ <107% |
| Rectum | D_0.1cc_ < 36.25Gy |
| PTV rectum | D0.1cc < 35Gy  <10.5Gy in the posterior rectal wall |
| Bladder | D0.1cc < 36.25Gy |
