# Supplementary figures and images for: Intrafractional stability of MR-guided online adaptive SBRT for prostate cancer
Source: Radiat Oncol. 2021 Sep 26;16:189. doi: 10.1186/s13014-021-01916-0 (PMC8474766; doi:10.1186/s13014-021-01916-0)

# D<sub>1cc</sub> Rectum

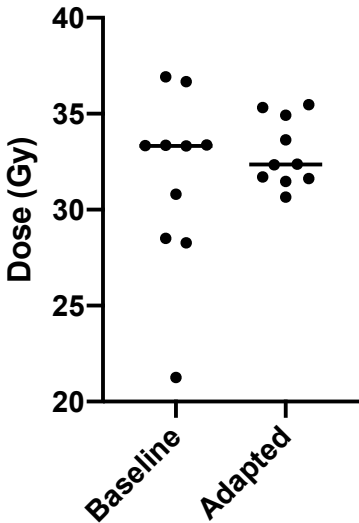

Supplement: Supplementary file 3 — Additional file 3: Fig. S1. D1ccrectum for the baseline plan on the MR of the day compared to the adapted plan. Bar = median. [file 13014_2021_1916_MOESM3_ESM.pdf]
